# In Vitro Evaluation of the Antifungal Activity of *Salvia Leriifolia* Leaf Hydroalcoholic Extract Compared to Nystatin and Fluconazole Against *Candida albicans*

**DOI:** 10.1002/cre2.70436

**Published:** 2026-08-19

**Authors:** Anahita Ghorbani, Negareh Salehabadi, Mina Ramezani, Iman Haghani, Majid Saeedi, Abolfazl Hosseinnataj, Maede Salehi

**Affiliations:** ^1^ Associate Professor, Department of Oral Medicine, Dental Research Center Mazandaran University of Medical Sciences Sari Iran; ^2^ Faculty of Dentistry, Mazandaran University of Medical Sciences Sari Iran; ^3^ Dentist, Mazandaran University of Medical Sciences Sari Iran; ^4^ Invasive Fungi Research Center Communicable Diseases Institute, Mazandaran University of Medical Sciences Sari Iran; ^5^ Department of Medical Mycology School of Medicine, Mazandaran University of Medical Sciences Sari Iran; ^6^ Professor, Department of Pharmaceutics Faculty of Pharmacy, Mazandaran University of Medical Sciences Sari Iran; ^7^ Assistant Professor, Department of Biostatistics Faculty of Health, Mazandaran University of Medical Sciences Sari Iran

**Keywords:** *Candida albicans*, fluconazole, hydroalcoholic extract, nystatin, *Salvia leriifolia*

## Abstract

**Objectives:**

With the rising incidence of fungal infections in recent years, *Candida albicans* has become the leading cause of oral fungal infections, ranging from superficial to systemic forms. The extensive use of antifungal medications has led to drug resistance and adverse effects, highlighting the growing need to explore medicinal plants. This study aimed to assess the antifungal activity of the hydroalcoholic extract of *Salvia leriifolia* leaves on laboratory strains of *Candida albicans*, comparing its effects with those of the standard antifungal drugs Nystatin and Fluconazole.

**Material and Methods:**

This experimental study used 20 confirmed *Candida albicans* isolates. Malt extract agar and RPMI‐1640 media were prepared according to standard protocols, and *Salvia leriifolia* extract was obtained via maceration. Fungal suspensions and stock solutions were prepared at defined concentrations, and MICs were determined using the microdilution broth method per CLSI‐M27‐A3 guidelines. Data were analyzed with SPSS.

**Results:**

*Salvia leriifolia* extract demonstrated antifungal activity against *Candida albicans*, with MIC values ranging from 500 to 1000 µg/mL and a geometric mean MIC of 901.3 µg/mL. In contrast, nystatin and fluconazole exhibited markedly lower MIC ranges (0.063–16 and 0.063–1 µg/mL, respectively) and geometric mean MICs of 0.297 and 0.308 µg/mL, respectively. Statistical analysis revealed significantly higher MIC values for the extract compared with both antifungal agents (*p* < 0.001, effect size = 0.87), whereas no significant difference was observed between nystatin and fluconazole (*p* = 0.638). Despite its lower potency, the extract showed moderate antifungal activity against *Candida albicans*.

**Conclusions:**

This study demonstrated that while the hydroalcoholic extract of *Salvia leriifolia* is less potent than nystatin and fluconazole, it is still capable of partially inhibiting the growth of *Candida albicans*. These results underscore the potential role of medicinal plants in the treatment of fungal infections, especially in cases involving drug resistance or undesirable side effects from conventional medications.

## 1 Introduction

Candidiasis represents a broad spectrum of opportunistic fungal infections, ranging from superficial mucosal involvement to severe systemic disease in certain individuals (Wingard 1995). It is recognized as the most common fungal infection affecting the oral cavity in humans. *Candida albicans* exists naturally within the oral flora of healthy individuals but can become pathogenic under certain conditions, leading to a variety of fungal infections (Lenka et al. 2020; Patel 2022). Numerous local and systemic factors contribute to the pathogenic shift of this organism, especially in immunocompromised individuals such as those living with HIV, patients undergoing prolonged antibiotic therapy, or denture wearers. In these groups, *Candida albicans* can transition from a commensal to a pathogenic state (Jacobsen 2023). Oral candidiasis may present in various clinical forms, affecting either localized areas or the entire oral cavity (Singh and Kumari 2024).

Several antifungal agents, including polyenes (e.g., nystatin), azole derivatives (e.g., fluconazole), and antiseptics like chlorhexidine, are commonly employed to treat *Candida albicans* infections. While these medications offer therapeutic benefits, they are not without adverse effects. Reports include bitter taste with nystatin tablets, allergic reactions to nystatin creams, and, in some cases, serious complications such as adrenal insufficiency, hepatic necrosis, and drug interactions (Chinsembu 2016; Hilal and Engelhardt 2007; Mollashahi et al. 2015). Fluconazole is a triazole antifungal used mainly against *Candida* infections. It works by inhibiting ergosterol synthesis, disrupting fungal cell membranes. However, resistance to fluconazole has increased due to genetic changes, efflux pump activation, and biofilm formation in fungi (Casalinuovo et al. 2004).

Due to these side effects, there is increasing interest among researchers in identifying effective, with fewer adverse effects; particularly those derived from medicinal plants (Baghalian and Naghdi Badi 2000). Medicinal herbs are considered valuable natural resources with therapeutic potential, and it is estimated that one‐third of pharmaceutical products originate from plant sources (Khalil et al. 2005). Among aromatic medicinal plants, members of the Lamiaceae family, especially the genus *Salvia* (sage), hold a prominent place (Pierozan et al. 2009).

*Salvia leriifolia* is a perennial herbaceous plant native to certain tropical regions of Iran (notably Khorasan and Semnan) and parts of Afghanistan (Rechinger 1982). Extracts and essential oils from various parts of this plant have demonstrated a wide range of therapeutic effects, including analgesic, anticonvulsant, antimicrobial, and antioxidant properties (Hosseinzadeh et al. 2009; Hosseinzadeh and Imenshahidi 1999; Farhoush et al. 2004; Hadad Khodaparast et al. 2006). Its aqueous leaf extract has sedative and muscle‐relaxant effects comparable to diazepam (Hosseinzadeh and Lary 2000), and its decoction shows anti‐inflammatory activity similar to diclofenac (Hosseinzadeh and Yavari 1999). Furthermore, both aqueous and alcoholic extracts of the seeds and leaves have shown protective effects against gastric ulcers in animal models (Hosseinzadeh et al. 2000).

Existing studies have also highlighted the antimicrobial and antifungal properties of essential oils and extracts from different *Salvia* species, particularly their inhibitory effects on *Streptococcus mutans*, *Streptococcus pyogenes*, *Streptococcus sanguis*, and *Actinomyces viscosus* (Abrishamchi et al. 2016), along with their antifungal activity. These findings suggest that *Salvia* species are promising candidates for further exploration in the context of antibacterial and antifungal applications (Hosseinzadeh et al. 2009).

Various species of the genus *Salvia*, including *Salvia officinalis* and *Salvia rhytidea*, have been investigated for their antifungal activity. *Salvia officinalis* is characterized by high levels of essential oils and phenolic compounds and has long been used in traditional medicine for its antiseptic and anti‐inflammatory properties, whereas *Salvia rhytidea* possesses simpler phytochemical constituents and more limited traditional applications (Ghadaksaz et al. 2025; Sookto et al. 2013; Zahabi et al. 2020). In contrast, *Salvia leriifolia* is rich in bioactive diterpenoids and phenolic compounds and has traditionally been used in Iranian medicine for wound healing and treatment of infections, which may explain its potentially stronger anti‐*Candida* activity compared with other *Salvia* species (Salimikia and Mirzania 2022).

Considering the side effects associated with chemical antifungal drugs, the documented anti‐inflammatory and antifungal properties of various *Salvia* species, and the absence of prior studies specifically investigating *Salvia leriifolia*, this study was designed to evaluate the in vitro antifungal effects of the hydroalcoholic leaf extract of *Salvia leriifolia* on *Candida albicans*, and to compare its efficacy with that of nystatin and fluconazole.

## 2 Methods

This study was an experimental‐laboratory study. A total of 20 *Candida albicans* isolates from the sample bank of the Invasive Fungal Research Center were cultured on Malt Extract Agar (MEA) for preparation. All samples had previously been identified using molecular methods, such as sequencing of the ITS1‐5.8S‐ITS gene region.

### 2.1 Preparation of Culture Media

A. **Malt Extract Agar Culture Medium**
  A specified amount of MEA powder (Blaeslee) was dissolved in distilled water according to the manufacturer's instructions, and the pH was adjusted to the standard level. The solution was autoclaved at 121°C for 15 min. Afterwards, it was poured into Petri dishes under sterile conditions, and once solidified, the media were stored at 4°C in the refrigerator to maintain their freshness and effectiveness for the following experiments.
B. **1640‐ Roswell Park Memorial Institute (RPMI) Culture Medium**
  According to the standard NCCLS‐M27‐A3 method, the 1640‐RPMI culture medium was used for diluting the drug stock and preparing the fungal suspension. To prepare this medium, 0.4 grams of 1640‐RPMI powder (Sigma‐Aldrich) and 34.53 grams of MOPS buffer (3‐(N‐morpholino) propanesulfonic acid) (Sigma‐Aldrich) were dissolved in 900 mL of distilled water and heated with a magnetic stirrer until fully dissolved. The pH was then adjusted to 7.0 at 25°C using sodium hydroxide (1 mol. lit^−1^). The final volume was adjusted to 1 liter by adding distilled water, and the medium was sterilized using 0.22‐micron filters. The medium was divided into 250‐mL portions in glass containers with screw caps and stored at 4°C. Prior to each use, the medium was carefully inspected for contamination.

### 2.2 Preparation of Yeast Suspension for the Microdilution Method

In accordance with the CLSI M27‐A3 guidelines, a suspension was first prepared from several (fresh) yeast colonies in 5 mL of sterile physiological saline. The concentration of *Candida* was measured using a spectrophotometer at a wavelength of 530 nm, aiming for a transmission value of 75–77%. The resulting stock suspension was then diluted 1:1000 with RPMI medium to prepare the inoculum suspension (National Committee for Clinical Laboratory Standards 2002).

### 2.3 Collection of *Salvia leriifolia* and Preparation of Extract

Fresh leaves of *Salvia leriifolia* were collected in April and May 2024, during the flowering season, which is considered the optimal time for harvest from areas surrounding Mashhad. After drying the leaves in the shade, they were ground into a powder. The extraction was performed by repeated soaking in 70% ethanol for 48 h, with the process repeated three times. The resulting extract was concentrated using a rotary evaporator under vacuum conditions. The concentrated extract was then placed in an oven at 35°C to ensure complete evaporation of the solvent. Finally, it was stored in a tightly sealed container at 4°C, protected from light, until further use for experiments.

### 2.4 Preparation of Antifungal Drugs

To prepare the drug stock solutions, a specific amount of the *Salvia leriifolia* extract, nystatin, and fluconazole was accurately weighed. For the *Salvia leriifolia* extract, 2 grams were transferred into a sterile microtube, and a defined amount of Dimethylsulfoxide (DMSO) was added to bring the final volume to 10 mL, resulting in a stock solution with a concentration of 200,000 µg/mL. For the nystatin stock solution, 320 mg of pure nystatin powder was weighed and dissolved by adding DMSO to a total volume of 100 mL of distilled water, achieving a concentration of 3200 µg/mL. Similarly, for fluconazole, 1280 mg of pure fluconazole powder was weighed and, after adding a specific amount of DMSO, the final volume was adjusted to 100 mL, resulting in a stock solution with a concentration of 12,800 µg/mL. To facilitate dissolution, all solutions were gently shaken or vortexed until completely dissolved. The prepared solutions were stored at 4°C, and for obtaining lower concentrations, they were serially diluted with 1640‐RPMI medium. Since the drug is diluted 1:100 with RPMI and the concentration is halved upon adding the fungal suspension, the concentration specified in the protocol for each drug was multiplied by 200, and the resulting number was converted to micrograms per 1 mL of the respective solvent. All steps were performed under sterile conditions to prevent contamination (National Committee for Clinical Laboratory Standards 2002).

### 2.5 Determination of Total Phenolic Content in Extract

The total phenolic content in plant extracts was determined by using Folin‐Ciocalteu method. Several concentrations of gallic acid solutions in methanol (10, 25, 50, and 75 μg/mL) were prepared. One mL of gallic acid of each concentration was added to test tube, then 5 mL of Folin‐Ciocalteu reagent (10%) and 4 mL of 7% Na_2_CO_3_ were added to get a total volume of 10 mL. The blue‐colored mixture was shaken well and incubated for 30 min at 40°C in a water bath. Then the absorbance was measured at 760 nm. All the experiments were carried out in triplicate. The average absorbance values obtained at different concentrations of gallic acid were used to plot the calibration curve.

Various concentrations of the *Salvia leriifolia* extract were prepared. Following the above procedure, the absorbance for each sample of extract was recorded. Total phenolic content of the extract was determined as mg gallic acid equivalents (GAE) per gram of sample in dry weight (mg/g). The total phenolic contents in all samples were calculated by using the formula:

*C* = *c V*/*m*, where.

*C* = total phenolic content mg GAE/g dry extract,

*c* = concentration of gallic acid obtained from calibration curve in mg/mL,

*V* = volume of extract in mL, and

*m* = mass of extract in grams (Genwali et al. 2013; Hosseinpoor et al. 2016).

The randman of extraction was 12.7 ± 0.45%. Total phenolic content in *Salvia leriifolia* was 41.12 ± 2.15 GAE/g, which was similar to Hosseinpoor et al's (2016) report.

### 2.6 Microdilution Broth Antifungal Susceptibility Test

This test was performed following the CLSI protocol. Initially, 100 µL of RPMI broth (prepared according to the protocol) was added to each well in a 96‐well plate, except for the first column from the left, where only 200 µL of the drug was added. The nystatin concentration in this column was set at 16 µg/mL, and the fluconazole concentration at 64 µg/mL (according to the CLSI‐M27‐A3 protocol). For the *Salvia leriifolia* extract, after several pilot experiments with different concentrations, a final concentration of 1 mg/mL was used in the first well. Using a multi‐sampler, 100 µL from the first column was transferred serially to the second column. After thoroughly mixing the solution with the RPMI medium by performing up‐and‐down movements, 100 µL was removed, and this process was repeated until the 11th column, with the final 100 µL discarded. This procedure generated serial dilutions of the drug in each row. Then, 100 µL of the prepared fungal suspension was added to all columns except the first. The 12th column, containing RPMI and fungal suspension, served as the positive control for growth, while the first column, without fungal suspension, was considered the negative control. The final volume in each well was 200 µL. The plates were incubated at 35°C for 24 h, and after incubation, the MIC values of the samples were recorded. This test was performed for 20 *Candida* isolates from a triplicate series (National Committee for Clinical Laboratory Standards 2002; Fothergill 2011).

### 2.7 Determination of Minimum Inhibitory Concentration (MIC) Values

In accordance with the CLSI‐M27‐A3 standard guidelines, MIC values were determined through visual assessment. The MIC is defined as the lowest drug concentration that results in approximately 50% inhibition of fungal growth after 24 h of incubation, as measured relative to a drug‐free growth control well (Hashemi et al. 2011). MIC values were determined by visual reading, following the guidelines outlined in the CLSI‐M27‐A3 standard protocol.

### 2.8 Data Analysis

Descriptive statistics such as mean, standard deviation, and median were employed to summarize the study variables. The Shapiro–Wilk test was applied to evaluate the normality of data distribution. Comparisons of *Candida albicans* levels between groups were conducted using the non‐parametric Kruskal–Wallis and Mann–Whitney tests. Statistical analyses were performed using SPSS software (version 22), with a significance threshold set at *p* < 0.05.

A post‐hoc power analysis was conducted to evaluate the adequacy of the sample size (*n* = 20 *Candida albicans* isolates) for detecting significant differences between the three antifungal agents. The analysis indicated that the current sample size provided sufficient power (>0.80) to detect statistically significant differences at an alpha level of 0.05, given the observed effect sizes.

### 2.9 Ethical Considerations

The present study was approved by the Medical Ethics Committee of Research Department of Mazandaran University of Medical Science (IR.MAZUMS.REC.1403.219). In this study, all ethical guidelines were followed, and all the collected data were used exclusively for this research.

## 3 Results

### 3.1 MIC Distribution Profiles

Descriptive data of MIC of three antifungal drugs nystatin, fluconazole, and *Salvia leriifolia* extract (SLE) were recorded on 20 *Candida albicans* isolates (Table 1).

**Table 1:** MIC distribution of nystatin, fluconazole, and Salvia leriifolia extract (µg/mL).

Parameter	SLE	Nystatin	Fluconazole
Range (Min–Max)	500–1000	0.063–16	0.063–1
Median (IQR)	1000 (1000–1000)	0.25 (0.125–0.50)	0.37 (0.25–0.50)
Geometric mean	901.3	0.297	0.308

### 3.2 Frequency Breakdown

- **SLE:** 17 isolates (85%) required 1000 µg mL^−^^1^ and 3 isolates (15%) 500 µg mL^−^^1^.
- **Nystatin:** 12 isolates (60%) were inhibited at ≤0.25 µg mL^−^^1^, 6 isolates (30%) at 0.5 µg mL^−^^1^, 2 isolates (10%) at ≥1 µg mL^−^^1^ (one at 16 µg mL^−^^1^).
- **Fluconazole:** 14 isolates (70%) were inhibited at ≤0.25 µg mL^−^^1^; the remaining 30% at 0.5–1 µg mL^−^^1^.

### 3.3 Inter‐Group Comparisons

The Kruskal–Wallis test demonstrated a highly significant difference in MIC ranks among the three agents (*H* = 40.92, *p* < 0.001). Results of pairwise Mann–Whitney *U*‐tests are shown in Table 2.

**Table 2:** Pairwise comparison of groups in terms of MIC value.

Type of antifungal		*U* value	*p* value	Effect size (*R*‐value)
SLE	Nystatin	5.65	<0.001*	0.87
SLE	Fluconazole	5.65	<0.001*	0.87
Nystatin	Fluconazole	0.47	0.638	0.25

* *p* < 0.05.

### 3.4 Susceptibility Classification (CLSI Break‐Points)

Using CLSI M27‐A3 interpretive criteria, fluconazole (susceptible ≤2 µg/mL) showed 100% susceptibility among isolates, as all MIC values were ≤1 µg/mL. No established CLSI breakpoints are available for nystatin; however, 95% of isolates exhibited MICs ≤1 µg/mL, indicating strong in vitro activity. For the plant extract *Salvia leriifolia*, which lacks CLSI‐defined breakpoints, MIC values ≥ 500 µg/mL were interpreted according to previously published phytochemical activity classifications as indicative of moderate‐to‐weak antifungal activity (MIC ≤ 100 µg/mL: Strong activity/100 µg/mL < MIC ≤ 625 µg/mL: Moderate activity/625 µg/mL < MIC: Weak activity) (Kuete and Efferth 2010).

Importantly, no isolate demonstrated reduced susceptibility to both fluconazole and nystatin, suggesting that the tested panel consisted of drug‐susceptible *Candida albicans* strains.

## 4 Discussion

The hydro‐alcoholic extract of *Salvia leriifolia* inhibited all *C. albicans* isolates yet was ~3 log‐units less potent than nystatin (polyene) or fluconazole (azole). Based on its MIC_90_ of 1000 µg/mL, the extract is considered to possess moderate antifungal activity. This level of activity is valuable; however, it does not support the use of the extract as a stand‐alone treatment (Kuete and Efferth 2010).

Numerous investigations have explored the antifungal effects of natural and plant‐based compounds against *Candida albicans*, with their MIC values summarized in the following table (Table 3).

**Table 3:** Reported MIC of some natural and plant‐based compounds against *Candida albicans*.

Botanical/compound	MIC against *C. albicans*	Reference
*Salvia leriifolia* (Hydroalcoholic Extract)	1000 µg/mL	Present study
*Salvia officinalis* (ethanolic extract)	25,000 µg/mL	Ghadaksaz et al. (2025)
*Salvia rhytidea* (methanolic fraction)	≥3125 µg/mL	Zahabi et al. (2020)
*Thymus vulgaris* (aqueous extract)	50,000 µg/mL (planktonic)	Oliveira et al. (2017)
*Cupressus sempervirens* (essential oil)	65–250 µg/mL (biofilm)	Hou and Huang (2024)
Curcumin	7.8–32.3 µg/mL (drug‐resistant strains)	Hajifathali et al. (2023)
Green‐tea catechins (EGCg)	Synergy with fluconazole at 64 µg/mL	Hirasawa (2004)

Our MIC is approximately 25‐fold lower than that reported for *S. officinalis* and at least three‐fold lower than *S. rhytidea*, indicating that *S. leriifolia* possesses a comparatively richer antifungal phytochemical profile within the genus (Ghadaksaz et al. 2025; Zahabi et al. 2020).

Essential oils of *Cupressus* spp. and curcumin achieve sub‐100 µg/mL MICs (an order of magnitude stronger than SLE), yet their clinical translation is hampered by volatility, cytotoxicity, or poor solubility (Hou and Huang 2024; Hajifathali et al. 2023). Consequently, SLE's moderate potency coupled with favorable safety (traditional use as herbal tea) may still warrant development as an adjunct.

Tea catechins enhance fluconazole and amphotericin B activity at sub‐MIC levels (Hirasawa 2004). Similar synergy experiments with SLE are rational next steps and could reduce the required extract concentration below the 100 µg/mL threshold considered pharmacologically attractive (Hsu et al. 2021).

Terpenoids such as rosmarinic and carnosic acids (abundant in *Salvia* spp.) disrupt ergosterol biosynthesis or generate oxidative stress in fungal membranes. Fractionation of SLE will clarify whether these compounds, or yet‐unidentified flavonoids, dominate the observed activity.

The findings of the present study have potential clinical relevance for dentistry. Although topical nystatin suspensions are effective in the management of oral candidiasis, their clinical use is often limited by poor patient compliance, while systemic azole antifungals are increasingly challenged by the emergence of resistant strains. In this context, a mucoadhesive gel or mouth rinse formulated with a standardized *S. laxiflora* leaf extract (SLE) in combination with a sub‐therapeutic dose of nystatin may represent a promising alternative. Such a formulation could reduce the selective pressure associated with azole use, improve patient acceptability through the herbal flavor and aroma of the extract, and provide a more accessible therapeutic option for populations with limited access to prescription antifungal agents. Nevertheless, translating the in vitro minimum inhibitory concentration (MIC; 0.1% w/v) into an effective in vivo dosage remains a significant challenge. Therefore, approaches such as combination therapy exploiting synergistic interactions (e.g., with fluconazole) or the incorporation of nano‐delivery systems may be necessary to achieve optimal therapeutic efficacy.

Despite these promising findings, several limitations should be acknowledged. The antifungal activity was evaluated using a planktonic model, which does not replicate the complexity of oral biofilms or account for physiological factors such as salivary flow and pH fluctuations. Furthermore, only *Candida albicans* was investigated, whereas the activity of the extract against non‐*albicans Candida* species and polymicrobial or mixed‐species cultures remains to be determined. The use of a crude plant extract also introduces potential variability in phytochemical composition, which may influence the reproducibility of the results; therefore, standardization using High‐Performance Liquid Chromatography is essential. In addition, fungicidal activity and time‐kill kinetics were not assessed, leaving the distinction between fungistatic and fungicidal effects unresolved.

Future investigations should focus on validating these findings under more clinically relevant conditions. Specifically, biofilm‐based models and time‐kill assays performed on clinically relevant substrates, such as acrylic discs, are needed to better evaluate the practical applicability of the extract. In addition, checkerboard assays with fractional inhibitory concentration index analysis should be conducted to quantify the potential synergistic interactions between SLE and established antifungal agents, including nystatin, fluconazole, and chlorhexidine. Bioassay‐guided fractionation followed by Liquid Chromatography–Mass Spectrometry analysis would facilitate the identification and enrichment of the bioactive compounds responsible for the observed antifungal activity. Finally, in vivo studies in animal models and humans, clinical trials are warranted to assess therapeutic efficacy, taste acceptability, and mucosal tolerability, while the development of nanocarrier‐based formulations, such as liposomal or muco‐penetrating delivery systems, may further improve the solubility, bioavailability, and retention of the extract within the oral cavity.

## 5 Conclusion

The hydro‐alcoholic leaf extract of *Salvia leriifolia* shows moderate, reproducible inhibition of *Candida albicans* (MIC = 1000 µg/mL) yet remains far less potent than nystatin and fluconazole. Within the *Salvia* genus it is, nonetheless, among the more active species and merits further investigation as a synergistic adjunct in topical antifungal therapy for oral candidiasis. Fully characterized phytochemical fractions, synergy studies, and translational work are required before clinical deployment.

## Author Contributions

**Maede Salehi** and **Anahita Ghorbani:** conceptualization, study design. **Negareh Salehabadi** and **Mina Ramezani:** data acquisition. **Abolfazl Hosseinnataj:** data interpretation, data analysis. **Negareh Salehabadi**, **Mina Ramezani**, **Majid Saeedi**, and **Iman Haghani:** writing – original draft. **Maede Salehi**, **Anahita Ghorbani**, **Negareh Salehabadi**, and **Iman Haghani:** writing – review and editing. **Maede Salehi**, **Anahita Ghorbani**, and **Majid Saeedi:** Final approval.

## Funding

The authors have nothing to report.

## Ethics Statement

The present study was approved by the Medical Ethics Committee of Research Department of Mazandaran University of Medical Science (IR.MAZUMS.REC.1403.219).

## Conflicts of Interest

The authors declare no conflicts of interest.

## Data Availability

The data that support the findings of this study are available from the corresponding author upon reasonable request.
